# 

**Published:** 1905-06

**Authors:** 


### ATLANTA JOURNAL-RECORD OF MEDICINE.

Successor to Atlanta Medical and Surgical Journal, Established 1855, and Southern Medical Record, Established 1870.

VOL. VII. ATLANTA, GA., JUNE, 1905. No. 3.

